# Phosphorylation of Pex11p does not regulate peroxisomal fission in the yeast *Hansenula polymorpha*

**DOI:** 10.1038/srep11493

**Published:** 2015-06-23

**Authors:** Ann S. Thomas, Arjen M. Krikken, Ida J. van der Klei, Chris P. Williams

**Affiliations:** 1 Molecular Cell Biology, Groningen Biomolecular Sciences and Biotechnology Institute, University of Groningen, the Netherlands.

## Abstract

Pex11p plays a crucial role in peroxisomal fission. Studies in *Saccharomyces cerevisiae* and *Pichia pastoris* indicated that Pex11p is activated by phosphorylation, which results in enhanced peroxisome proliferation. In *S. cerevisiae* but not in *P. pastoris*, Pex11p phosphorylation was shown to regulate the protein’s trafficking to peroxisomes. However, phosphorylation of PpPex11p was proposed to influence its interaction with Fis1p, another component of the organellar fission machinery. Here, we have examined the role of Pex11p phosphorylation in the yeast *Hansenula polymorpha*. Employing mass spectrometry, we demonstrate that HpPex11p is also phosphorylated on a Serine residue present at a similar position to that of ScPex11p and PpPex11p. Furthermore, through the use of mutants designed to mimic both phosphorylated and unphosphorylated forms of HpPex11p, we have investigated the role of this post-translational modification. Our data demonstrate that mutations to the phosphorylation site do not disturb the function of Pex11p in peroxisomal fission, nor do they alter the localization of Pex11p. Also, no effect on peroxisome inheritance was observed. Taken together, these data lead us to conclude that peroxisomal fission in *H. polymorpha* is not modulated by phosphorylation of Pex11p.

Peroxisomes are single membrane bound organelles that house a variety of metabolic processes. Common functions include detoxification of hydrogen peroxide and beta-oxidation of fatty acids. In humans, failure of peroxisome function can lead to severe lethal disorders, such as Zellweger syndrome, demonstrating their importance in cellular metabolism and health^1^.

Depending on cellular demands, peroxisomes may vary in size, number and content. In yeast the main mode of peroxisome proliferation is fission, a process that involves three steps: 1) organelle elongation, 2) membrane constriction and 3) membrane scission^2^. Pex11p is a peroxisomal membrane protein (PMP) that is a key player in the first step of peroxisome fission^2^. Pex11p was first identified as a factor involved in fission in the yeast *Saccharomyces cerevisiae*, and it is the most abundant peroxisomal membrane protein^3^. In all organisms studied to date, deletion of *PEX11* results in a reduction in peroxisome numbers whereas its overexpression stimulates peroxisome proliferation^4^,^5^,^6^,^7^. Previous work from our group and others established that Pex11p stimulates membrane elongation through the action of an amphipathic helix, which upon interaction with the peroxisomal membrane imparts a curvature^6^,^8^. Pex11 proteins can also interact with other proteins of the organelle fission machinery, such as Fis1p and Mff^9^,^10^,^11^ and current models propose that the concerted action of these proteins exerts control over the division of peroxisomes^12^. Although Pex11p is indispensable for peroxisome fission^13^, how it is triggered to participate in this process, or how it achieves interaction with other components of the fission machinery, is still not well understood.

Previously, Pex11p was shown to be phosphorylated in two yeast species: *S. cerevisiae* and *Pichia pastoris*^11^,^14^. In both cases, it was observed that phosphorylation of Pex11p is crucial for organelle proliferation, because strains producing mutant variants mimicking constitutively phosphorylated Pex11p showed enhanced peroxisome proliferation while mutants mimicking unphosphorylated Pex11p were characterized by reduced peroxisome numbers. Although it was established that Pex11p phosphorylation is important for peroxisome proliferation in both yeast species, different molecular mechanisms were proposed. In *S. cerevisiae*, phosphorylation was shown to influence trafficking of Pex11p from the endoplasmic reticulum (ER) to peroxisomes, whereas in *P. pastoris* Pex11p trafficking was shown to be independent of phosphorylation. Instead in this organism, phosphorylation was shown to be important for binding of Pex11p to Fis1p. In order to gain further insight into the function of Pex11p phosphorylation, we studied Pex11p in the yeast *Hansenula polymorpha*. Our data indicate that this protein is indeed phosphorylated, however unlike in *S. cerevisiae* and *P. pastoris*, this modification appears not to play a significant role in peroxisome proliferation or Pex11p localization.

## Results and Discussion

### HpPex11p is phosphorylated at a similar site to its counterparts in *S. cerevisiae* and *P. pastoris*

**Figure 1:**
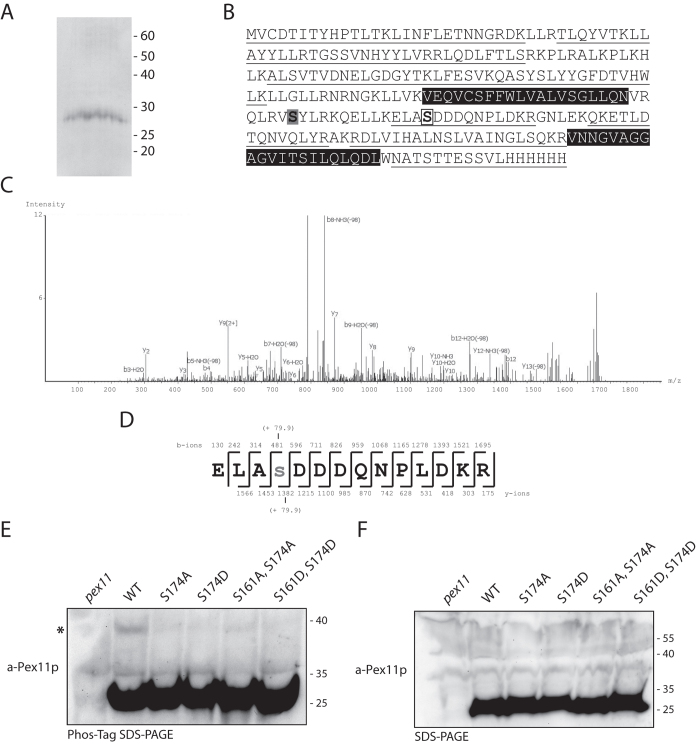
HpPex11p is phosphorylated on Serine 174. (**A**) Coomassie stained SDS-PAGE gel showing a band at ~30 kDa, corresponding to Pex11-His_6_. Numbers indicate molecular weight in kDa. (**B**) Sequence coverage obtained for Pex11-His_6_ from MS analysis. Peptides identified in this approach are underlined. Both Serine 174 (open box) and Serine 161 (grey shaded box) are indicated. Black shading depicts the predicted transmembrane domains. (**C**) nLC-MS/MS analysis of the modified 171-ELASDDDQNPLDKR-184 peptide identified using MS. (**D**) Sequence of the 171- ELASDDDQNPLDKR-184 peptide, demonstrating that the additional 79.9 Daltons, corresponding to a phosphate group, is present on Serine 174. Indicated are the b and y ions, as well the modified Serine residue (lower case). (**E** and **F**) Phos-tag^TM^ SDS-PAGE (**E**) or SDS-PAGE (**F**) and western blotting analysis of lysates from *pex11Δ* cells (*pex11*) or *pex11Δ* cells expressing WT or mutant forms of Pex11p. Blots were probed with antibodies raised against Pex11p. Equal amounts of protein were loaded per lane. The modified form of Pex11p visible in WT cells is denoted with an asterisk.

In order to investigate a potential role of Pex11p phosphorylation in *H. polymorpha*, we purified the protein and analysed it using mass spectrometry (MS). For this, we employed a version of Pex11p complete with C-terminal His_6_ tag. This version of Pex11p fully complements the *pex11Δ* deletion strain (Supplementary Figure S1A and B), demonstrating that it is functional. Pex11p-His_6_ was purified from an organellar pellet (Fig. 1A) and subsequent MS analysis resulted in efficient sequence coverage of Pex11p (Fig. 1B). Furthermore, we identified two peptides that corresponded to the residues 171-ELASDDDQNPLDKR-184. One displayed the predicted molecular mass of approximately 1615 Dalton, whereas the other displayed a molecular mass gain of 79.9 Dalton, which is indicative of the presence of a phosphate group. Peptide sequencing of this peptide (Fig. 1C,D) revealed that the additional 79.9 Dalton was present on the Serine at position 174, strongly suggesting that Pex11p is phosphorylated at this Serine residue.

*H. polymorpha* Pex11p contains 47 residues that can be modified by a phosphate group (18 Serines, 17 Threonines and 12 Tyrosines) and of these 47, only 5 were not covered in our MS analysis (Fig. 1B). One of these residues, Serine 161, is the closest Serine residue to the identified phosphorylation site at position 174. The other 4 residues not detectable in our MS analysis are present in the predicted transmembrane domains of Pex11p, which makes them unlikely phosphorylation sites.

Unlike the situation in *P. pastoris*, where the phosphorylated form of Pex11p is visible using SDS-PAGE and western blotting^11^, we were not able to observe any modified forms of Pex11p using similar techniques. Therefore, we employed Phos-Tag^TM^ acrylamide gels to investigate Pex11p phosphorylation further. This approach utilises the Phos-Tag^TM^, a small molecule that, when incorporated into SDS-PAGE, specifically binds to and inhibits the mobility of phosphorylated proteins^15^. In such an analysis, phosphorylated proteins display a decreased mobility on SDS-PAGE, compared to unphosphorylated forms. Using this approach (Fig. 1E), we see a minor portion of wild type (WT) Pex11p with reduced mobility compared to the major Pex11p species, suggesting that a very small portion of Pex11p is phosphorylated. This species is not visible when using standard SDS-PAGE analysis (Fig. 1F).

Taken together, our data indicate that like ScPex11p and PpPex11p, HpPex11p is also phosphorylated on a Serine residue. This residue is present at a similar position to the Pex11p phosphorylation sites reported in *S. cerevisiae* and *P. pastoris* (Supplementary Figure S1C).

### Mutations to the HpPex11p phosphorylation site do not affect peroxisome proliferation

Next, we generated mutant versions of Pex11p, to lock the protein in a constitutively unphosphorylated (Pex11 S174A) or phosphorylated (Pex11 S174D) form. Both mutant versions as well as a WT control gene were introduced into a *pex11* deletion strain. Strains were constructed that either co-produced the fluorescent peroxisomal membrane marker PMP47-GFP or the matrix marker GFP-SKL. First, we investigated the phosphorylation status of these mutants using Phos-Tag^TM^ SDS-PAGE (Fig. 1E) and observed that the modified form of Pex11p is greatly reduced when Serine 174 is mutated, strongly suggesting that introduction of mutations at position 174 inhibits Pex11p phosphorylation.

**Figure 2:**
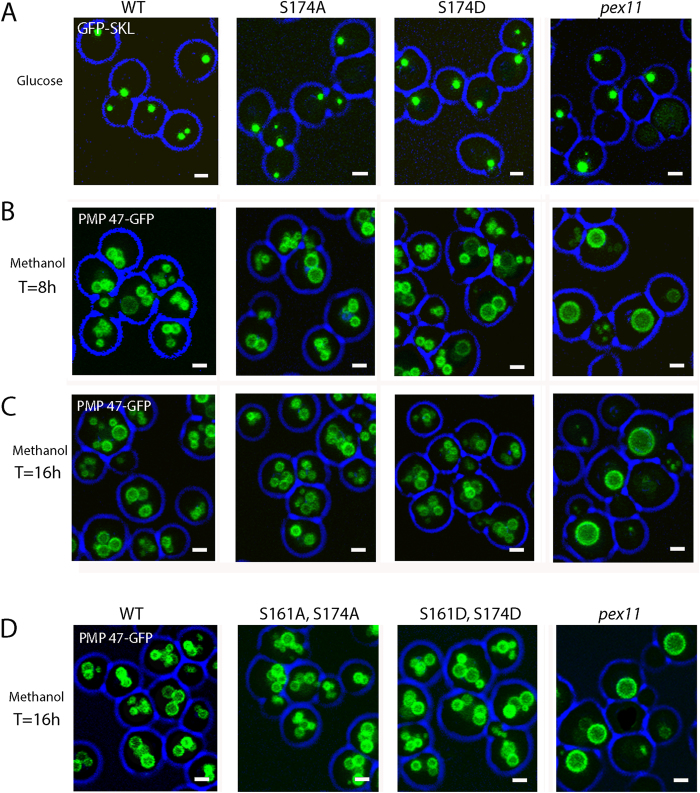
Phosphorylation of HpPex11p does not affect peroxisome abundance. Fluorescence microscopy images of *pex11Δ* cells grown on glucose (**A**) or methanol (**B-D**), using confocal laser scanning microscopy (CLSM). GFP-SKL was used to mark peroxisomes in glucose-grown cells, whereas PMP47-GFP was used to mark peroxisomes in methanol-grown cells. Besides fluorescent markers, cells produced WT or phosphorylation single mutant (**A-C**) or double mutant (**D**) forms of Pex11p, as indicated above panels. All scale bars represent 1 μm.

**Figure 3:**
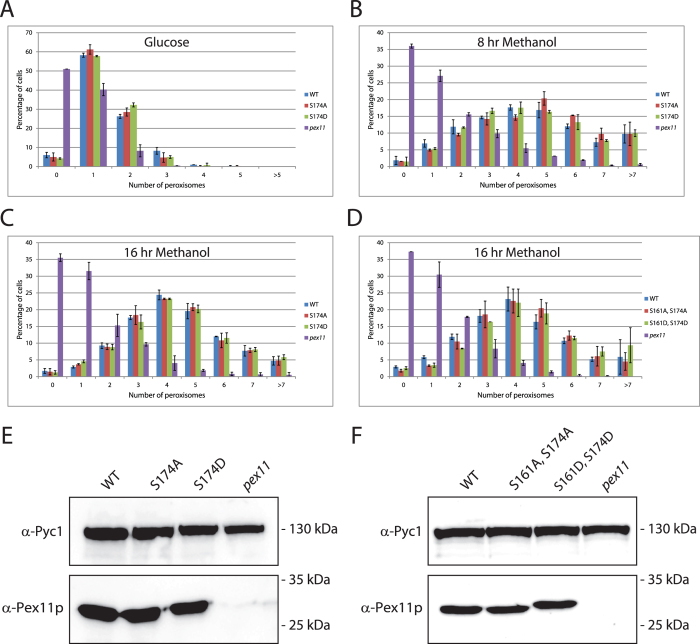
Quantitative analysis of peroxisome numbers in WT and HpPex11p phosphorylation mutant strains. (**A-D**) Quantification of peroxisome numbers from images represented in Fig. 2A–D. For each experiment, at least 600 cells were counted per strain. Error bars represent standard deviation between two separate experiments. (**E** and **F**) Western blots to compare protein levels between WT and phosphorylation single mutant strains (**E**), or phosphorylation double mutant strains (**F**). In the case of mutants S174D and S161D, S174D, the introduction of an extra negative charge results in a mobility shift. Cells were grown for 16 hours on medium containing methanol. Blots were probed with antibodies raised against Pyruvate carboxylase-1 (Pyc1; loading control) or Pex11p. Equal amounts of protein were loaded per lane.

Next, fluorescence microscopy analysis (Fig. 2A–C; Fig. 3A–C) revealed that peroxisome numbers were comparable between strains expressing WT or mutant versions of HpPex11p, independent of whether cells were grown on glucose (Fig. 2A) or methanol (Fig. 2B,C). Western blotting revealed that Pex11p levels were comparable among strains (Fig. 3E). These findings suggest that phosphorylation of Serine 174 has no significant effect on peroxisome proliferation in glucose or methanol grown *H. polymorpha*.

Since the peptide containing Serine 161 could not be analysed during our MS analysis, we wanted to rule out the possibility that this residue may either be phosphorylated at a very low level, or that it may function as the phosphorylation site in the absence of Serine 174, as was reported for ScPex11p^14^. Therefore, double mutants of Pex11p were created such that both Serine residues at positions 161 and 174 were mutated either to Alanine (Pex11 S161A, S174A) or to Aspartic acid (Pex11 S161D, S174D). Phos-Tag^TM^ SDS-PAGE analysis again confirmed that Pex11p phosphorylation was inhibited in these mutants (Fig. 1E).

Next, by employing fluorescence microscopy, we observed that, as in the single mutant strains, no significant differences in peroxisome numbers between WT or double mutant versions of Pex11p could be observed (Figs 2D and 3D). Pex11p levels in these mutants were comparable to WT (Fig. 3F). Thorough imaging and quantitative analysis of all strains imply that HpPex11p is capable of fulfilling its function in peroxisomal fission independently of its phosphorylation status.

### Mutations in the phosphorylation site do not alter HpPex11p localization

**Figure 4:**
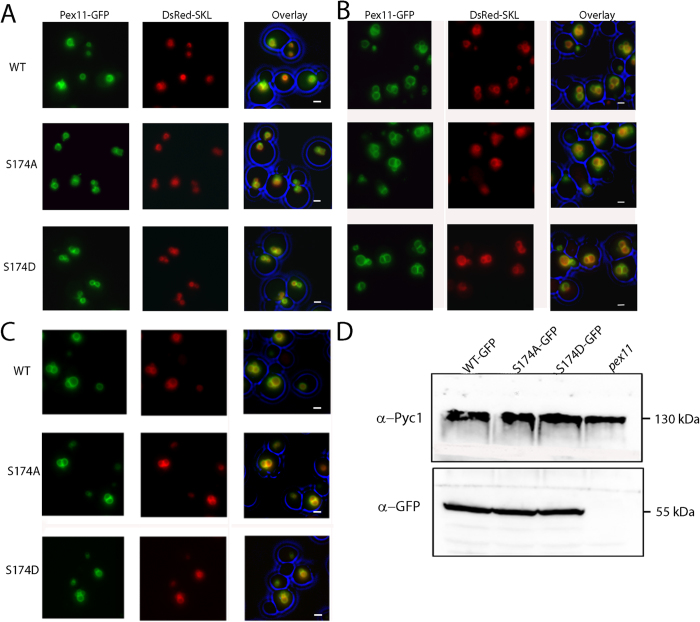
Phosphorylation of HpPex11p does not affect localization of the protein. (**A–C**) Wide field fluorescence microscopy images of *pex11Δ* cells grown on methanol for 4h (**A**), 8h (**B**) or 16 h (**C**). Besides DsRed-SKL, cells also produced GFP fusions of the Pex11 proteins. All scale bars represent 1 μm. (**D**) Western blot to compare protein levels of Pex11 S174A-GFP or Pex11 S174D-GFP with WT Pex11-GFP. Cells were grown for 16 hours on methanol. Blots were probed with antibodies raised against Pyc1 or GFP.

To analyse if Pex11p phosphorylation influences the subcellular location of Pex11p, strains were constructed producing WT and mutant versions of Pex11p C-terminally tagged with GFP. Cells were pre-cultivated on glucose and subsequently shifted to medium containing methanol, to induced peroxisome proliferation. At 4 and 8 hours after the shift, we observed that like WT Pex11-GFP, both mutant forms co-localise with DsRed-SKL, introduced into the strains to mark the peroxisomal matrix (Fig. 4A–C). Western blotting revealed that the protein levels were comparable among strains (Fig. 4D). These data suggest that phosphorylation does not regulate HpPex11p localisation.

### Peroxisome inheritance remains unperturbed in HpPex11p phosphorylation mutants

**Figure 5:**
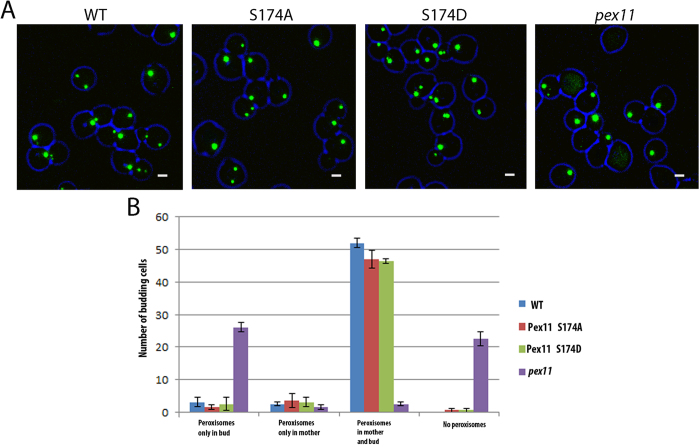
Phosphorylation of HpPex11p does not govern peroxisome inheritance. (**A**) Fluorescence microscopy images of glucose-grown *pex11Δ* cells using CLSM. Apart from the fluorescent peroxisomal matrix marker (GFP-SKL), cells produced WT or phosphorylation single mutant forms of Pex11p. All scale bars represent 1 μm. (**B**) Quantitative analysis of the images represented in (**A**) to show peroxisome distribution between mother and daughter cells. For all strains, at least 50 budding cells were counted. Error bars represent the standard deviation between two individual experiments.

It was shown previously that *H. polymorpha pex11Δ* cells display a peroxisome retention defect during growth on glucose^13^. The single peroxisome present in a cell migrates to the daughter cell upon budding, leaving the mother cell devoid of peroxisomes. To study if phosphorylation has a potential role in this process, budding cells of WT and Pex11p phosphorylation mutants producing GFP-SKL as peroxisomal marker were examined by fluorescence microscopy (Fig. 5A). We observed that peroxisome distribution over mother cells and buds is similar in WT and both phosphorylation mutants (Fig. 5B). This observation suggests that Pex11p phosphorylation does not affect peroxisome retention in *H. polymorpha*.

### Concluding remarks

Pex11p is critical for peroxisome division on both glucose and methanol. To the best of our knowledge, loss of Pex11p function cannot be rescued by other proteins. This not only reiterates the pivotal role of this protein in peroxisomal fission, but also the need to understand how it may control the fission event. Our data demonstrate that phospho-mimicking mutant versions of *H. polymorpha* Pex11p behave identically to the WT protein, data that lead us to conclude that phosphorylation does not play a vital role in peroxisomal fission, inheritance or in Pex11p localisation. This raises the possibility that alternate mechanisms may exist to modulate the role of HpPex11p in these processes. Identifying such mechanisms would provide valuable insight into the molecular mechanisms underlying peroxisomal inheritance and fission.

## Methods

### Strains and cultivation conditions

The *H. polymorpha* strains used in this study are listed in Table 1. All strains used here are derived from the *pex11Δ* parent strain^13^. *H. polymorpha* cells were grown in batch cultures at 37 °C on mineral media^16^ supplemented with 0.25% glucose or 0.5% methanol as carbon source and 0.25% ammonium sulphate as nitrogen source. Leucine, when required, was added to a final concentration of 30 μg/ml. For growth on plates, YPD (1% yeast extract, 1% peptone and 1% glucose) media was supplemented with 2% agar. Resistant transformants were selected using 100 μg/ml zeocin or 100 μg/ml nourseothricin (Invitrogen). For cloning purposes, *Escherichia coli DH5a* was used as the host for propagation of plasmids. Cells were grown at 37 ^o^C in Luria broth supplemented with ampicillin (100 ug/ml).

To analyse growth in batch cultures, the optical densities at 600 nm were measured at different time points. Three independent cultures were used for each strain.

### Construction of plasmids

The plasmids and oligonucleotides used in this study are listed in Tables 2 and 3, respectively. pHIPZ17-Nia, the plasmid containing the Pex11 promoter (P_Pex11_) was constructed as follows: to isolate the PEX11 promoter a fragment of 0.9 kb upstream the *PEX11* gene was amplified using primer pex11-1 and pex11-2 and genomic DNA as template. The resulting fragment was digested with *Hind*III and *Not*I and ligated in *Hind*III-*Not*I digested pHIPZ4-Nia^17^ resulting in vector pHIPZ17-Nia.

To obtain WT PEX11 under control of the endogenous promoter (P_*PEX11*_), plasmid pHIPZ4-Pex11 was digested with *Hind*III and *Sal*I and the resulting fragment ligated into *Hind*III-*Sal*I digested pHIPZ17-Nia, resulting in the vector pCW297. To obtain WT PEX11, complete with C-terminal His6 tag, under control of the endogenous promoter (pCW323), polymerase chain reaction (PCR) was performed on plasmid pCW297 using primers Pex11 HIII and Pex11-His SalI and the product was digested with *Hind*III and *Sal*I and ligated into *Hind*III-*Sal*I digested pHIPZ17-Nia. Both these vectors were linearized with *Nsi*I prior to transformation into *H. polymorpha* cells.

A plasmid containing the *PEX11* promoter and the *PEX11* gene fused to GFP was constructed as follows: first the *PEX11* promoter was isolated by PCR using primer Pex11-A and Pex11-E using genomic *H. polymorpha* DNA as a template. The resulting fragment was digested with *Hind*III and *Psp*XI and ligated into pSNA10^18^, resulting in plasmid pAMK64. Subsequently the PEX11 gene was isolated by PCR using primer PEX11-F and PEX11-D using *H. polymorpha* DNA as a template. This fragment was restricted with *Sal*I and *Bgl*II and was ligated into plasmid pAMK64 restricted with *Bgl*II and *Psp*XI, resulting in plasmid pAMK65. This plasmid was linearized with *BstAP*I for integration into *H. polymorpha* cells.

All point mutants were produced using the QuickChange Site-Directed Mutagenesis Kit (Agilent) and all constructs produced by PCR were confirmed by sequencing. Plasmids to produce PEX11 point mutants, under control of the PEX11 promoter were created using pCPW297 as template together with the mutagenic primer pairs described in Table 3. Plasmids were linearized using *Nsi*I prior to integration into the genome of *H. polymorpha* cells. Point mutant versions of PEX11-GFP were created using pAMK65 as template and the primer pairs listed in Table 3. Plasmids were linearized using *BstAP*I for integration into *H. polymorpha* cells.

The plasmid bearing GFP-SKL, downstream of the constitutive promoter TEF1, was constructed as follows: PCR was performed using primers GFPN5_Fw and GFPN5_Rev using plasmid pHIPX5 GFP-SKL as template. The resulting product was digested with *Not*I and *Xba*I and ligated into *Not*I-*Xba*I digested pHIPN5^19^, producing the plasmid pHIPN5 GFP-SKL. Next, pHIPX7 GFP-SKL^20^ was digested with *Not*I and *Bam*HI, to obtain the TEF1 promoter and this fragment was ligated into pHIPN5 GFP-SKL, to produce pHIPN7 GFPSKL. This plasmid was linearized using the enzyme *Stu*I prior to transformation into *H. polymorpha* cells.

The plasmid pHIPN4 DsRed-SKL^21^ was linearized using *Nsi*I prior to transformation into *H. polymorpha* cells.

The plasmid pMCE07^21^, bearing the C-terminal region of PMP47 fused to GFP, was linearized with *Mun*I prior to transformation into *H. polymorpha* cells.

### Cell fractionation and purification of HpPex11p

*H. polymorpha* cells containing the Pex11p-His_6_ expression cassette were grown for 10 hours on mineral medium containing 0.5% methanol (in order to induce Pex11-His_6_ production). Cells were harvested by centrifugation (10 min, 12,000x g at room temperature (RT). Protoplasts were prepared using Zymolyase (Brunschwig Chemie, Amsterdam, the Netherlands) and homogenized using a Potter homogenizer. The resulting cell lysate was centrifuged twice at 3,000 x g (10 min, 4 °C). The post nuclear supernatant (PNS) was centrifuged at 30,000 x g (30 min, 4 ^o^C) to separate the supernatant and membrane pellet fractions. This organelle fraction was used as the starting material to extract Pex11-His_6_. The pellet was resuspended in 50 mM Tris, pH 7.4, 300 mM NaCl, 3 mM beta mercaptoethanol, 0.5% IGEPAL CA-630, 10 mM imidazole, phosphatase inhibitor cocktail (Sigma Aldrich), PMSF (Sigma) and 1% glycerol. Supernatant and pellet fractions from this mixture were separated by ultracentrifugation (15 min at 20,0000 x g at 4 ^o^C) and the soluble fraction was incubated with Ni-NTA beads (QIAGEN) for 1 hour at 4 ^o^C with shaking. The column was extensively washed with wash buffer and bound proteins were eluted with elution buffer (50 mM Tris-HCL, 300 mM NaCl, 3 mM beta mercaptoethanol, 330 mM Imidazole, 1% glycerol, 0.1% IGEPAL CA-630). The resulting purified fraction was subjected to sodium dodecyl sulphate polyacrylamide gel electrophoresis (SDS-PAGE) and coomassie staining.

### Mass spectrometric analysis

The coomassie stained Pex11-His_6_ band was excised from gel and submitted for MS analysis. The gel fragment was washed with 100 mM ammonium bicarbonate and acetonitrile and suspended in 100 mM ammonium bicarbonate. Proteolytic treatment was performed using the proteases Trypsin, Chymotrypsin and GluC (Promega) in order to determine the best protease for detection of the phosphorylated peptides. Peptides were extracted with 75% acetonitrile and 25% (5% formic acid in water) and analysed by nano liquid chromatography-tandem mass spectrometry (nLC-MS/MS)^22^. MS data was analysed with PEAKS 7.0 software (Bioinformatics Solutions Inc.).

### Biochemical techniques

Cell extracts of trichloroacetic acid treated cells were prepared for SDS-PAGE as detailed previously^23^. Phos-Tag^TM^ acrylamide was obtained from Wako Chemicals and Phos-Tag^TM^ containing SDS-PAGE gels were prepared according to the manufacturer’s instructions. Equal volumes of lysates were loaded per lane and gels were subjected to western blot analysis. Blots were probed with rabbit polyclonal antiserum against Pex11p, pyruvate carboxylase (Pyc1) or mouse monoclonal antiserum against GFP (Santa Cruz Biotechnology, sc-9996). Secondary antibodies conjugated to horseradish peroxidase were used for detection. Blots were scanned using a densitometer (Biorad).

### Fluorescence microscopy

All images were made at room temperature using a 100 × 1.30 NA Plan Neofluar objective.

Wide-field images were made using a Zeiss Axioscope A1 fluorescence microscope (Carl Zeiss, Sliedrecht, The Netherlands). Images were taken using a Coolsnap HQ2 digital camera and Micro Manager software. A 470/40 nm bandpass excitation filter, a 495 nm dichromatic mirror and a 525/50 nm bandpass emission filter was used to visualize the GFP signal. DsRed fluorescence was visualized with a 546/12 nm bandpass excitation filter, a 560 nm dichromatic mirror and a 575/640 nm bandpass emission filter.

Confocal images were acquired with a confocal microscope (LSM 510; Carl Zeiss), equipped with photomultiplier tubes (Hamamatsu Photonics) and Zen 2009 software. GFP fluorescence was analysed following excitation of cells with a 488-nm Argon ion laser (Lasos), and emission was detected using a 500–550 nm band-pass emission filter.

Image analysis was done using Adobe Photoshop CS4 and Image J, and Adobe Illustrator was used for figure preparation. Unless indicated otherwise, the intensity maximum and minimum were set equally for all imaged indicated within a single figure panel, thus allowing direct fluorescence intensity comparison between different strains.

### Quantification

For quantification of peroxisome numbers in *H. polymorpha*, yeast cells were detected with a custom-made plugin for ImageJ. Using the brightfield image slices as input, the cells are approximated by a 3-dimensional ellipsoid. For the detection of peroxisomes, another plugin was developed. This plugin (available on request) uses the data from the fluorescent channel and was designed to parse clumps of peroxisomes. For this, clumps of connected peroxisomes are isolated on each z-slice. Next, the outline of each peroxisome clump is described by a chain of interconnected nodes. Concave regions in the chain indicate a transition between two adjacent peroxisomes. The convex regions between these transitions are then used to fit circles. Finally, the data from all the z-slices are combined, and the separate peroxisomes are described as spheres.

For analysis of peroxisome numbers using PMP47-GFP, Z- stacks were made of arbitrarily chosen fields. Strains were grown in duplicates and quantification was done on 4 images per culture, wherein each image contained at least 100 cells each. Cells are expressed in percentage and error bars indicate the standard deviation between two cultures of the same strain.

When GFP-SKL was used for quantification of peroxisome numbers, images were acquired as previously described, and peroxisomes were counted manually. Strains were grown in duplicates and at least 200 cells were counted for each strain.

For quantitative analysis of peroxisome inheritance, pictures of budding cells were selected randomly as a stack in bright field as well as fluorescence mode. Z-stacks were made containing 10 optical slices of 0.9 μm thickness in order to cover the entire cell. The Z-axis spacing was set to 0.6 μm, to avoid missing any fluorescent signal.

The Zeiss LSM IMAGE BROWSER software was used to determine the cross-sectional area of the mother and bud cell. Assuming yeast cells to be spherical, bud volume was determined as a percentage of the mother cell, wherein the volume of the mother cell was set to 100%. Only cells with a bud volume lower than 25% were considered as bud cells for the analysis. Not less than 50 cells per strain was counted for the quantification.

### In silico analysis

Multiple sequence alignments of protein sequences were prepared using T-Coffee (http://tcoffee.vital-it.ch/apps/tcoffee/do:regular) and boxshade (http://www.ch.embnet.org/software/BOX_form.html)

## Additional Information

**How to cite this article**: Thomas, A. S. *et al.* Phosphorylation of Pex11p does not regulate peroxisomal fission in the yeast *Hansenula polymorpha*. *Sci. Rep.* **5**, 11493; doi: 10.1038/srep11493 (2015).

## Supplementary Material

Supplementary Information

## Figures and Tables

**Table 1:** *Hansenula polymorpha* strains used in this study.

Strains	Characteristics	Reference
*pex11* + Pex11-His_6_	*PEX11* deletion strain with pCW323	This study
*pex11* + PMP47-GFP	*PEX11* deletion strain with pMCE7	This study
*pex11* + WT PEX11 + PMP47-GFP	*PEX11* deletion strain with pCW297 and pMCE7	This study
*pex11* + PEX11 S174A + PMP47-GFP	*PEX11* deletion strain with pANN003 and pMCE7	This study
*pex11* + PEX11 S174D + PMP47-GFP	*PEX11* deletion strain with pANN004 and pMCE7	This study
*pex11* + WT PEX11 + GFP- SKL	*PEX11* deletion strain with pCW297 and pHIPN7 GFP-SKL	This study
*pex11* + PEX11 S174A + GFP- SKL	*PEX11* deletion strain with pANN003 and pHIPN7 GFP-SKL	This study
*pex11* + PEX11 S174D + GFP- SKL	*PEX11* deletion strain with pANN004 and pHIPN7 GFP-SKL	This study
*pex11* + PEX11 S164A,S174A + PMP47-GFP	*PEX11* deletion strain with pANN005 and pMCE7	This study
*pex11* + PEX11 S164D, S174D + PMP47-GFP	*PEX11* deletion strain with pANN006 and pMCE7	This study
*pex11* + PEX11-GFP + DsRed-SKL	*PEX11* deletion strain with pAMK64 and pHIPN4 DsRed-SKL	This study
*pex11* + PEX11 S174A-GFP + DsRed-SKL	*PEX11* deletion strain with pANN007 and pHIPN4 DsRed-SKL	This study
*pex11* + PEX11 S174D-GFP + DsRed-SKL	*PEX11* deletion strain with pANN008 and pHIPN4 DsRed-SKL	This study

**Table 2:** Plasmids used in this study.

Plasmid	Description	Reference
pCW297	Pex11 expressed from endogenous promoter, contains Zeo^R^	This study
pCW323	Pex11-His_6_ expressed from endogenous promoter, contains Zeo^R^	This study
pANN003	Pex11S174A phos mutant expressed from endogenous promoter, contains Zeo^R^	This study
pANN004	Pex11S174D phos mutant expressed from endogenous promoter, contains Zeo^R^	This study
pANN005	Pex11S161, 174A phos double mutant expressed from endogenous promoter, contains Zeo^R^	This study
pANN006	Pex11S161, 174D phos double mutant expressed from endogenous promoter, contains Zeo^R^	This study
pAMK65	Pex11 expressed from endogenous promoter, with C-terminal GFP fusion, contains Zeo^R^	This study
pANN007	Pex11S174A expressed from endogenous promoter, with C-terminal GFP fusion, contains Zeo^R^	This study
pANN008	Pex11S174D expressed from endogenous promoter, with C-terminal GFP fusion, contains Zeo^R^	This study
pHIPN7GFP-SKL	GFP-SKL expressed from P_TEF_ promoter, contains Nat^R^	This study
pHIPN4 DsRed-SKL	DsRed-SKL expressed from the alcohol oxidase promoter, contains Nat^R^	21
pMCE7	C- terminal region of PMP47-GFP fused to GFP, contains Zeo^R^	21

**Table 3:** Oligonucleotides used in this study.

Primer	Sequence (5’to 3’)
Pex11-1	ATAAGAATGCGGCCGCGTGGACTGCTACGAGACATT
Pex11-2	CCCAAGCTTATAACTGTCTGTCTGTCCC
Pex11 HIII	GCGCGCGCAAGCTTATGGTTTGCGACACGATAAC
Pex11-His Sal1	GCGCGCGCGTCGACTCAGTGATGGTGATGGTGATGTAGCACAGAAGACTCGGTC
Pex11-A	CCCAAGCTTGTGGACTGCTACGAGACATT
Pex11-E	AGAGCTCGAGGATAACTGTCTGTCTGTCCC
Pex11-F	ACGCGTCGACATGGTTTGCGACACGATAAC
Pex11-D	GGAAGATCTTAGCACAGAAGACTCGG
Pex11 174 S-A CS	CTCAAAGAGCTGGCCGCGGATGACGACCAGAAC
Pex11 174 S-A NCS	GTTCTGGTCGTCATCCGCGGCCAGCTCTTTGAG
Pex11 174 S-D CS	CTCAAAGAGCTGGCCGACGATGACGACCAGAACCCACTG
Pex11 174 S-D NCS	CAGTGGGTTCTGGTCGTCATCGTCGGCCAGCTCTTTGAG
Pex11 161 S-A CS	GTCAGACAACTCAGAGTGGCGTATCTGAGAAAACAAGAG
Pex11 161 S-A NCS	CTCTTGTTTTCTCAGATACGCCACTCTGAGTTGTCTGAC
Pex11 161 S-D CS	GTCAGACAACTCAGAGTGGACTATCTGAGAAAACAAGAGCTGCTCAAA
Pex11 161 S-D NCS	TTTGAGCAGCTCTTGTTTTCTCAGATAGTCCACTCTGAGTTGTCTGAC
PEX11 INT (F)	CGAAGACCGCACAATGGAGTTG
Pex11-10 (R)	CGCAACAAGAGCCTTCA
Pex11-9 (F)	CGGATACGCAATGGACA
GFPN5_Fw	ATAAGAATGCGGCCGCAATTCTTTCACCGCCCCACGCC
GFPN5_Rev	CTAGTCTAGATTACAGCTTCGACTTGTACAGC
