# Supplementary material for: Phosphorylation of Pex11p does not regulate peroxisomal fission in the yeast Hansenula polymorpha
Source: Sci Rep. 2015 Jun 23;5:11493. doi: 10.1038/srep11493 (PMC4477233; doi:10.1038/srep11493)
Supplement: Supplementary Information [file srep11493-s1.pdf]

Supplementary information

Phosphorylation of Pex11p does not regulate peroxisomal fission in the yeast *Hansenula polymorpha*

Ann S. Thomas, Arjen M. Krikken, Ida J. van der Klei and Chris P. Williams#

Molecular Cell Biology, Groningen Biomolecular Sciences and Biotechnology Institute, University of Groningen, the Netherlands.

#Address for correspondence: Chris Williams, Molecular Cell Biology, Groningen Biomolecular Sciences and Biotechnology Institute, University of Groningen, Nijenborgh 7, 9747AG Groningen, The Netherlands

Tel: +31 50 3632188

Fax: +31 50 3638280

Email: c.p.williams@rug.nl

Supplementary Figure S1. Pex11-His$_6$ fully complements the *pex11Δ* strain. (A) Glucose grown *pex11Δ* cells or *pex11Δ* cells expressing WT or His$_6$ tagged forms of Pex11p, under control of the PEX11 promoter were shifted to methanol containing media and the optical density (OD) at 600nm was measured at the indicated time points. Graphs represent the mean ± standard deviation of three separate experiments. (B) Western blot showing that Pex11-His$_6$ levels are comparable to Pex11p levels in WT. Cells were grown for 16 h on methanol. Blots were probed with antibodies raised against Pyc1 or Pex11p. (C) Sequence alignment of *Hansenula polymorpha* (Hp) Pex11p together with *Saccharomyces cerevisiae* (Sc) and *Pichia Pastoris* (Pp) Pex11p. Black shading indicates identity and grey shading similarity when present in at least two of the three sequences. The phosphorylation site(s) in *H. polymorpha* (solid triangle), *S. cerevisiae* (black asterisks) and *P. pastoris* (grey asterisk) are indicated.

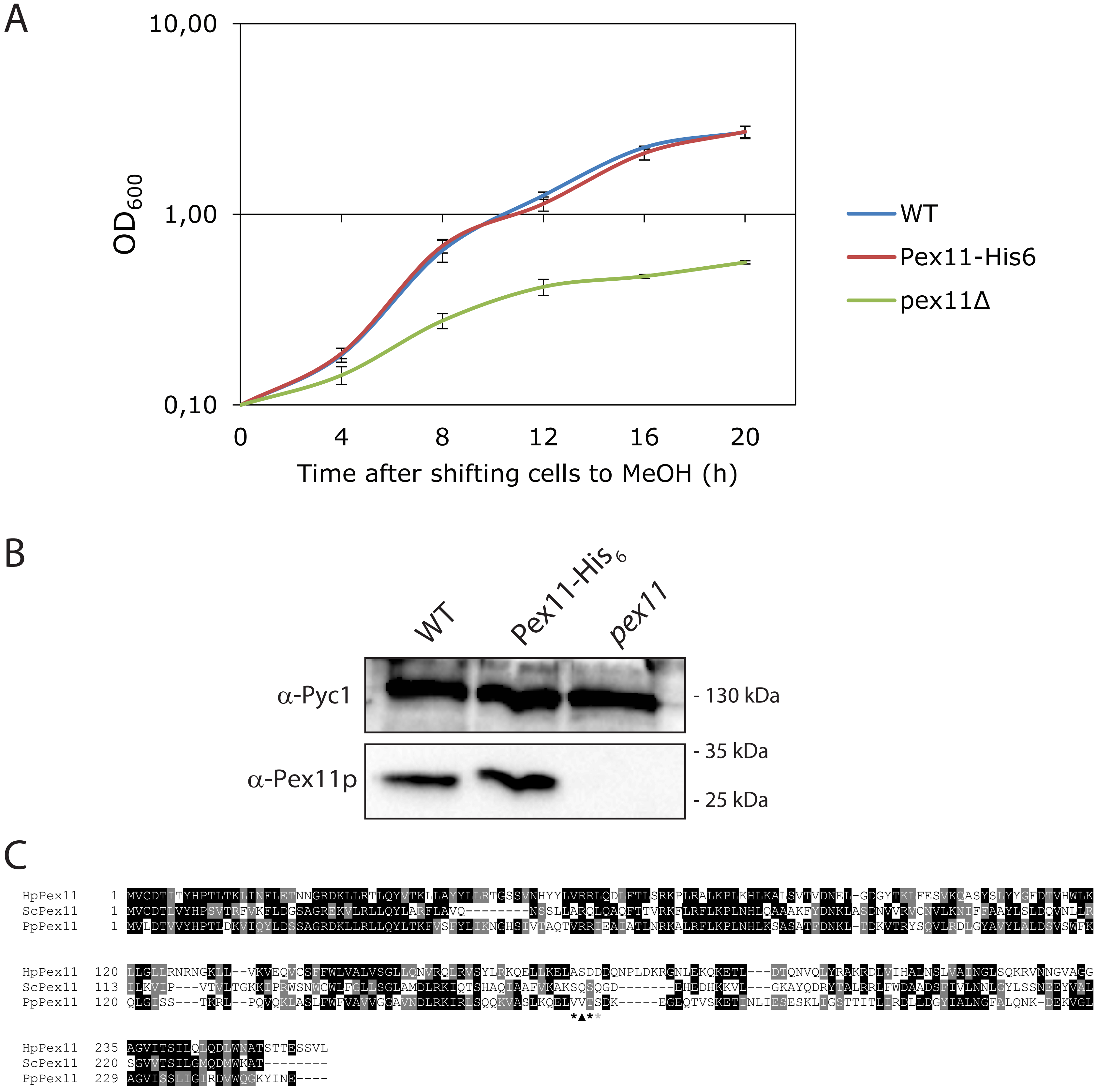
A
10,00
1,00
0,10
OD600
0
4
8
12
16
20
Time after shifting cells to MeOH (h)
WT
Pex11-His6
pex11Δ
B
WT
Pex11-His6
pex11
α-Pyc1
α-Pex11p
- 130 kDa
- 35 kDa
- 25 kDa
C
HpPex11 1 MVCDTITYHPTLTKLINFLETNNGRDKLLRTLQYVTKLLAYYLLRTGSSVNHYYLVRRLQDLFTLSRKPLRALKPLKHLKALSVTVDNEL-GDGYTKLFESVKQASYSLYYGFDTVHWLK
ScPex11 1 MVCDTLVYHPSVTRFVKFLDGSAGREKVLRLLQYLARFLAVQ---------NSSLLARQLQAQFTTVRKFLRFLKPLNHLQAAAKFYDNKLASDNVVRVCNVLKNIFFAAYLSLDQVNLLR
PpPex11 1 MVLDTVVYHPTLDKVIQYLDSSAGRDKLLRLLQYLTKFVSFYLIKNGHSIVTAQTVRRIEAIATLNRKALRFLKPLNHLKSASATFDNKL-TDKVTRYSQVLRDLGYAVYLALDSVSWFK
HpPex11 120 LLGLLRNRNGKLL--VKVEQVCSFFWLVALVSGLLQNVRQLRVSYLRKQELLKELASDDDQNPLDKRGNLEKQKETL---DTQNVQLYRAKRDLVIHALNSLVAINGLSQKRVNNGVAGG
ScPex11 113 ILKVIP---VTVLTGKKIPRWSNWCWLFGLLSGLAMDLRKIQTSHAQIAAFVKAKSQSQGD--------EHEDHKKVL---GKAYQDRYTALRRLFWDAADSFIVLNNLGYLSSNEEYVAL
PpPex11 120 QLGISS---TKRL--PQVQKLASLFWFVAVVGGAVNDLRKIRLSQQKVASLKQELVVTSDK-----EGEQTVSKETINLIESESKLIGSTTITLIRDLLDGYIALNGFALQNK-DEKVGL
*▲**
HpPex11 235 AGVITSILQLQDLWNATSTTESSVL
ScPex11 220 SGVVTSILGMQDMWKAT--------
PpPex11 229 AGVISSLIGIRDVWQGKYINE----
